# Age and Number of Surgeries Increase Risk for Complications in Polytrauma Patients with Operative Maxillofacial Fractures

**DOI:** 10.29252/wjps.7.3.307

**Published:** 2018-09

**Authors:** Shadi Lalezari, Christine Lee, Keyianoosh Z. Paydar, Ashkaun Shaterian

**Affiliations:** Department of Plastic Surgery, University of California, Irvine; Orange, CA, USA

**Keywords:** Poly-trauma, Facial fracture, Complication

## Abstract

**BACKGROUND:**

Poly-trauma patients often sustain complex head/neck injuries requiring prolonged hospitalizations and multiple operations. Few studies have evaluated the associated injury patterns and risk factors for poor clinical outcomes.

**METHODS:**

Consecutive poly-trauma patients with operative maxillofacial fractures treated at a level 1 trauma medical center between 1995 and 2013 were evaluated. Concomitant head/neck injuries to identify potential injury patterns were numerated. Lastly, a multivariate analysis was performed to determine independent risk factors for complications during the acute hospitalization period.

**RESULTS:**

Totally, 232 poly-trauma patients presented with operative maxillofacial fractures, while 38.8% of patients had a secondary maxillofacial fracture, 16.4% had intracranial hemorrhage, 23.7% had skull fractures, and 12.1% had spinal fractures. The rate of complication during admission was 28.3%. Multivariate analysis revealed advanced patient age and increased number of operations to predict the rate of complication. Patients requiring more than one operation had a 1.8-fold increase in complication rate (*p*<0.01) and older patients had a 4.5% increase in complication rate (*p*<0.05) for every year of increased age.

**CONCLUSION:**

Poly-trauma patients have a high incidence of secondary maxillofacial fractures, concomitant head/neck injury, and inpatient complication rate. Knowledge of associated injury patterns can help increased awareness and can guide physician decision-making to avoid missed/delayed injuries.

## INTRODUCTION

Poly-trauma patients sustain complex head/neck injuries often requiring prolonged hospitalizations, numerous operations, and coordinated multi-specialty care.^1^ With an estimated mortality of 18-23%,^2^^-^^6^ this population is characterized by high rates of morbidity and complication.^2^ Several studies have explored the poly-trauma populations, however, few have evaluated the injury profile of associated head/neck injuries and the risk factors for complication rates and poor clinical outcomes.^2^^-^^7^

Maxillofacial trauma patients can present with specific injury patterns. Maxillofacial injuries are often associated with high rates of secondary head/neck injury following trauma, however, are often overlooked during initial assessments. Vles *et al.* found that 14.3% of trauma patients with a delayed diagnosis had facial fractures.^3^ Further, numerous authors have revealed that facial trauma can be associated with secondary maxillofacial fractures and secondary injuries that may not readily be identified including pulmonary, spinal, ocular, and head injuries.^4^^-^^7^

Inadequate clinical assessment, truncated radiologic workup, delayed presentation of injury, and poor mental status can contribute to missed injuries/diagnoses.^8^^,^^9^ To this end, identifying potential injury patterns can help guide evaluation and treatment to prevent missed/delayed diagnosis in this vulnerable patient population. Poly-trauma patients often present with maxillofacial fractures and are known to have increased rates of complication during hospitalization.^7^^,^^10^^-^^12^

Complication rates for trauma patients admitted to the ICU are estimated at 17.0-31.2%, whereas complication rates following traumatic facial fractures range from 12.0-50.3%.^9^^,^^13^^-^^17^ Etiology of complications in the trauma population is multifactorial and dependent on multi-organ injury, the severity of injury, prolonged hospital stay, numerous invasive diagnostic/therapeutic procedures, amongst others.^14^^,^^15^ While, few authors have evaluated risk factors related to complications, a comprehensive analysis evaluating patient and surgery-related factors has yet to be explored.

Accordingly, the objective of the current study was (i) to identify the relationship between operative maxillofacial fractures and the presence of secondary head/neck injuries and (ii) to identify patient and surgical risk factors for complication. Increased awareness of risk factors, associated injuries, and complications following maxillofacial trauma can help improve clinical outcomes for this complex patient population.

## MATERIAL AND METHODS

In the current study, a retrospective, cross-sectional analysis was performed to investigate consecutive polytrauma patients treated at a Level 1 trauma medical center.Polytraumapatients included a subset of patients requiring inpatient hospitalization for multisystem injury. A cohort of patients presenting with maxillofacial fractures using ICD9 diagnosis codes was identified. The patients who underwent surgical intervention for maxillofacial fractures by the plastic surgery department using CPT codes and chart review were sub-selected. They were cross-compared with ICD9 codes for secondary spinal fracture, intracranial hemorrhage, and skull fracture. 

Patient charts were evaluated for variables related to socio-demographics, surgery, injury presentation, and complication rate. Next, the associated injury patterns concomitant with maxillofacial fracture were numerated to identify potential injury patterns. Finally, a multivariate analysis was performed to evaluate sociodemographic and surgery related variables to identify the risk factors for increased complications.

Patients who underwent operative repair for maxillofacial injuries were identified based on ICD9 and CPT codes. Patient charts were evaluated for sociodemographic variables including age, gender, race/ethnicity, comorbidities, toxicology screen, and insurance type. Race was categorized as Caucasian, Asian, African American, Hispanic, and Other. Insurance types were categorized as Private, Medicare, Medicaid, self-pay, and no insurance. We evaluated variables related to the trauma including the mechanism of injury, number of injuries sustained, need for ICU admission, and number of surgeries performed. Finally, the complication rates occurring during the acute hospitalization period were evaluated.

Given the high incidence of complications following poly-trauma cited in literature,^16^^,^^17^ a multivariate analysis was performed to identify independent risk factors for complications. Patients who suffered operative maxillofacial injuries were numerated and compared to identify secondary head/neck injuries including spinal fractures, ICH, and skull fractures. Next, univariate and multivariate regression analyses were performed to identify the variables influencing complication rates during the acute hospitalization period. First, a univariate analysis was performed to identify predictors of complications across each categorical or continuous variable using ANOVA. 

After identifying the significant predictors of complication on univariate analysis, these variables were included into a multivariate linear regression analysis and identified the predictors of complication while simultaneously controlling for confounding variables. Statistical significance was set at *p*-values<0.05 with all tests two-sided. All statistical analyses were conducted with IBM SPSS software. Approval for this study was obtained from the Human Research Protection (HRP) and Institutional Review Board at the University of California, Irvine Medical Center.

## RESULTS

Between 1995 and 2013, 232 poly-trauma patients received operative management of their maxillofacial fractures by the plastic surgery department. Patient characteristics are summarized in Table 1. With respect to patient demographics, the majority of patients included in the study were male (77.1%), and the mean age was 32.3 years. After stratifying for patient race/ethnicity, 58.2% of patients were found to be Caucasian, 16.4% were Hispanic, 8.6% were Asian, 3.0% were Black, and 13.9% were “other/unknown”. Next, the insurance types were evaluated and found 34.1% had private insurance, 8.6% had Medicare, 6.5% had Medicaid, 25.4% were self-pay, and 25.4% unknown. 2Among them, 6.7% had known comorbidities at the time of their injury and of the 84.1% of the patients tested for toxicology, and 27.8% had positive toxicology screens on admission.

**Table 1:** Patients’ characteristics

**Patients’ characteristics**	
Age (average +/- SD)	32.3 +/- 18
Sex	
Male	77.1%
Female	22.9%
Race/ethnicity	
Caucasian	58.2%
Hispanic	16.4%
Asian	8.1%
Black	3.2%
Other	13.7%
Mechanism of injury	
Assault	26.6%
Auto vs. Pedestrian	11.7%
Motor vehicle collision	53.2%
Other/Unknown	8.5%
Comorbidities	
Yes	26.6%
No/Unknown	73.4%
Toxicology screen	
Positive	23.4%
Negative	60.0%
Not Tested	16.0%
Insurance	
Private	34.1%
Medicare	8.5%
Medical	6.4%
Self pay	25.5%
Unknown	25.5%
Number of injuries	
1-3	18.1%
4-6	35.1%
7-10	28.7%
>10	18.1%
Number of procedures performed	
1-2	43.6%
3-4	28.7%
>4	27.7%
ICU Admission rate	47.9%
Complication rate	28.7%

The incidence of maxillofacial fractures was numerated and found 232 operative facial fractures occurred with 90 fractures occurring in association with a secondary maxillofacial fracture (Table 2). Next, the mechanisms of injury were evaluated and found that motor vehicle collision was most common cause (53.0%), followed by assault (26.7%), automobile vs. pedestrian (11.6%) and an unknown or other mechanism (8.6%). Almost half of the patients included in our study, 47.8% required admission to the ICU.

**Table 2:** Patients’ injuries

**Injuries**	
**Total cases (n)**	232
Primary fracture (n)	
Mandible	115
Nasal	84
Orbit	96
Zygoma	45
Secondary injury (n)	
Intracranial Hemorrhage	38
Maxillofacial fracture	90
Skull fracture	55
Spine fracture	28

An analysis of the injuries sustained by these patients revealed that 18.1% of patients had 1-3 injuries, 34.9% had 4-6 injuries, 28.9% had 7-10 injuries, and 18.1% had greater than 10 injuries. Next, the operative management of the injuries rendered and found 43.5% of patients required 1-2 surgeries, 28.9% required 3-4 surgeries, and 27.6% of patients required more than 4 surgeries.

To identify potential fracture patterns, the incidence of secondary maxillofacial fracture was evaluated. As shown in Table 2, 90 patients (38.8%) sustained a secondary maxillofacial fracture. The association between operative maxillofacial fracture and secondary skull fracture, IHC, and spinal fracture was explored. Here, 23.7% of patients suffered a secondary skull fracture, 16.4% suffered an intracranial hemorrhage, and another 12.1% of patients had a coexistent spine fracture. 

The overall complication rate during the acute hospitalization period for the study cohort was 28.3%, with sepsis, respiratory failure, and pneumonia/pnuemonitis being the most common. After controlling for sociodemographic and injury/disease related variables in our multivariate modeling (Table 3), the number of operations and patient age was found to independently predict complications. The patients who required more than one operation were found to have a 1.8-fold increase in their complication rate (OR1.804, *p*<0.01). With respect to patient age, patients had a 4.5% increase in complications for every year of older age (OR1.045, *p*<0.05).The remaining patient demographics (i.e. race, insurance, comorbidity, toxicology screen), mechanism of injury, or number of injuries were not significant predictors of complications.

**Table 3:** Multivariate analysis identifying risk factors for complications

**Variable**	**OR (CI)**	**P value**
Age (year)	1.045 (1.005-1.087)	<0.05
Number of operations (<1 vs. >1)	1.8 (1.296-2.511)	<0.05
Sex		NS
Race/Ethnicity		NS
Mechanism of injury		NS
Comorbidities		NS
Toxicology screen		NS
Insurance		NS
ICU admission		NS
Number of injuries		NS

## DISCUSSION

Maxillofacial fracture is a common finding in poly-trauma patients and one that often presents with multiple concomitant injuries and significant morbidity, mortality, and complication.^16^ In the current study, we evaluated poly-trauma patients who underwent operative repair of facial fractures and found a high rate of secondary maxillofacial fractures (38.8%). Associated injuries also included intracranial hemorrhage (16.4%), skull fractures (23.7%), and spinal fractures (12.1%). 

We found a complication rate of 28.3% during the acute hospitalization period and revealed that patients who underwent multiple operations, as well as those who presented with advanced age, were more likely to suffer a complication (*p*<0.05). The presence of medical comorbidities or number of injuries incurred failed to influence complication rates. Maxillofacial fractures often present with an incidence of associated life-threatening injuries.^17^

Similar to previous reports,^18^^-^^21^ we found associated injuries including intracranial hemorrhage (16.4%), skull fractures (23.7%), and spinal fractures (12.1%). Rates of associated injuries vary from 1.9-19% for skull fractures,^19^^,^^22^^-^^25^ 11.0% to 43.7% for brain injury,^9^^,^^24^ and 0.8-24% for cervical spine injury.^27^^,^^28^ The relative lack of protection of the head and neck in these high-impact settings make the face vulnerable to injury.^26^ The coexistence of head and neck injury following maxillofacial fractures is likely secondary to the proximity of the cranial/spinal bones to the facial bones and the ability to transmit direct traumatic impact to the cranium and spine via sutural attachments.^24^^,^^27^

Further, these significant injuries can be missed or delayed. Kloss *et al.* found that ~3% of patients with facial fractures and no neurological abnormalities exhibited intracranial hemorrhage on computed tomography (CT) scans.^28^ Similarly, Davis *et al.* found diagnosis of cervical spine injury was missed or delayed in 4.6% of patients-often due to inadequate or misread cervical spine imaging.^29^ Recognizing the frequency of multiple injuries and identifying maxillofacial fracture patterns can help alert physicians to potential concomitant injuries that may otherwise be under-evaluated.

In the present study, we found a complication rate of 28.3% in the acute hospitalization period for poly-trauma patients presenting with operative maxillofacial fractures. Previous studies have estimated the complication rate of trauma patients admitted to the ICU to be 17.0- 31.2%,^16^^,^^17^ whereas, others have found complication rates of 12.0-50.3% for traumatic facial fractures.^9^^,^^19^^,^^20^ Studies have shown age, gender, traumatic CNS injury, chronic alcohol use, the type of intensive care unit, and number of operations to influence complication rates.^16^^,^^17^^,^^21^^,^^30^^,^^31^

In the current study, we found patients requiring more than one operation had a 1.8-fold increase in complication rate. This may reflect added anesthesia risks, risks for DVT/PE inherent to surgery, infectious risks associated with incision,^21^ inflammatory response induced by surgical intervention itself, and others. While each operative intervention may be necessary and responsible for the decrease in mortality of trauma patients, clinicians must evaluate the necessity of additional operations, its timing, and the possibility of performing surgery on a delayed basis. 

Advanced age is a demographic variable that influences numerous medical and surgical outcomes. In this study, we found age to be an independent predictor of complication during the acute hospitalization period. While mortality rates have improved overall, Lonner *et al.* revealed elderly patients are at increased risk of dying following poly-trauma after finding patients >80 years had a 46% inpatient mortality rate vs. 10% in patients 66 to 79 years old.^32^ Similar previous studies have shown the associated increased mortality, length of stay, and cost of treatment with advancing patient age.^33^^-^^35^

This likely reflects increased comorbidities of the elder population,^36^ decreased functional reserve for recovery, and multisystem changes that occur with age. Ultimately, a more conservative approach for older patients may prove beneficial as would delay non-urgent surgical intervention. Further, physicians must be informed about the risks associated with advanced age and encouraged to be proactive with planning of services, such as rehabilitation, to improve clinical outcomes.^37^

This study has several limitations. First, this study was a retrospective cross-sectional study, and was therefore subject to potential uncontrolled and unmeasured biases. Although we were able to identify risk factors and predictors of complication, the study was unable to establish a causal-effect relationship. Further, the present study included data from a single institution in a single urban setting. A different geographical area may represent different patient populations, etiologies for traumatic injury, and access to trauma centers. Further, our study did not evaluate timing of surgical intervention or the duration of operations, which may have affected complication rates. Despite these limitations, our study was able to evaluate the incidence of secondary maxillofacial and head/neck injuries and identified risk factors for complications to help guide clinical decision-making.

Poly-trauma patients have a high incidence of secondary maxillofacial fractures, concomitant head/neck injury, and inpatient complication rate. Knowledge of associated injuries is necessary to comprehensively evaluate, treat, and avoid missed/delayed injuries. Older age and number of operations can contribute to higher complications during the acute hospitalization period. Delaying non-urgent operative intervention after the initial hospitalization period as well as providing more conservative management for older patients may decrease complication rates and improve outcomes.

## CONFLICT OF INTEREST

The authors declare no conflict of interest.
